# The clinical observation of ciprofol versus propofol anesthesia in hysteroscopic electrosurgical resection of submucosal myomas

**DOI:** 10.3389/fmed.2026.1829147

**Published:** 2026-04-28

**Authors:** Haibing Li, Tong Li, Yong Guo, Xiaoshu Zheng, Bin Lin

**Affiliations:** 1 Department of Anesthesiology, Shanghai First Maternity and Infant Hospital, School of Medicine, Tongji University, Shanghai, China; 2 Key Laboratory of Anesthesiology and Resuscitation, Huazhong University of Science and Technology, Ministry of Education, Wuhan, China; 3 Department of Biochemistry and Molecular Biology, Pennsylvania State University, University Park, PA, United States; 4 Department of Critical Care Medicine, Shanghai Jiao Tong University Affiliated Sixth People’s Hospital, Shanghai, China; 5 Department of Gynecology, Shanghai First Maternity and Infant Hospital, School of Medicine, Tongji University, Shanghai, China

**Keywords:** alfentanil, anesthesia, ciprofol, hysteroscope, propofol, resection

## Abstract

**Background:**

To evaluate the clinical efficacy of ciprofol versus propofol combined with alfentanil for anesthesia in patients undergoing hysteroscopic transcervical resection of submucosal myomas.

**Methods:**

A total of 208 patients scheduled for hysteroscopic transcervical resection of submucosal myomas were randomly allocated into ciprofol (group C) and propofol (group P) groups after exclusion. All patients received intravenous alfentanil 10 μg/kg before induction. The group C received intravenous ciprofol 0.4 mg/kg, while the group P received intravenous propofol 2.0 mg/kg. After anesthesia induction, a laryngeal mask airway was inserted. Anesthesia was maintained with a continuous infusion of ciprofol 0.8 mg/(kg·h) or propofol 5 mg/(kg·h) using a micro-infusion pump until the end of surgery. Mean arterial pressure (MAP), heart rate (HR), and bispectral index (BIS) were recorded at the following time points: Pre-anesthesia (T0), after anesthesia induction (T1), during cervical dilation (T2), during electrosurgical resection (T3), and at the end of surgery (T4). Total dosage of study drug, recovery time, orientation recovery time, visual analogue scale (VAS) scores for uterine contraction pain after awakening, and adverse events were recorded.

**Results:**

Intraoperative decreases in MAP and HR were significantly greater in the group P than in the group C (*p* < 0.05). The total anesthetic dosage was significantly higher in the group P (*p* < 0.05). Recovery time and orientation recovery time were longer in the group C compared with the group P (*p* < 0.05). VAS scores at 5 and 15 min after awakening were significantly lower in the ciprofol group (*p* < 0.05). The incidence of injection pain was much higher in Group P than that in Group C (*p* < 0.001). The rate of respiratory inhibition was significantly lower in Group C than that in Group P (*p* < 0.05).

**Conclusion:**

Ciprofol exhibits comparable efficacy to that of propofol. It has good analgesic activity, more stable hemodynamics, and minimal respiratory disturbance, less injection pain rate in hysteroscopic transcervical resection of submucosal myoma.

## 1 Introduction

Hysteroscopic transcervical resection of submucosal myoma is characterized by minimal surgical trauma and rapid postoperative recovery (1). However, intraoperative cervical dilation, uterine traction, and negative pressure suction may induce abdominal pain, nausea, and agitation, leading to patient discomfort or even life-threatening complications (2). The choice of anesthetic technique is closely associated with procedural safety, patient comfort, and postoperative outcomes (3). Total intravenous anesthesia using propofol combined with opioids is commonly applied in clinical practice (4). Propofol has advantages such as rapid onset, good controllability, and fast recovery, but is also associated with injection pain, respiratory depression, and hemodynamic instability (5). Ciprofol is a novel short-acting intravenous anesthetic and a *γ*-aminobutyric acid type A (GABA_A_) receptor agonist, characterized by rapid onset and recovery, reduced injection pain, high potency, and a wide therapeutic window. Compared with propofol, ciprofol is associated with a lower incidence of respiratory and cardiovascular adverse events, suggesting potential clinical advantages.

This study aimed to compare the anesthetic and analgesic effects, hemodynamic changes, and adverse reactions of ciprofol versus propofol in hysteroscopic transcervical resection of submucosal myoma, thereby providing clinical evidence for anesthetic management. In this study, alfentanil was administered as a standardized analgesic component in both groups to ensure a consistent antinociceptive baseline, allowing for a more accurate comparison of the sedative and adverse effect profiles of ciprofol and propofol.

## 2 Materials and methods

### 2.1 Participants

Patients undergoing hysteroscopic transcervical resection of submucosal myoma from June 2025 to December 2025 were enrolled. Inclusion criteria were ASA physical status I ~ II, age 23 ~ 41 years, body weight 50 ~ 80 kg, and body mass index (BMI) 18 ~ 31 kg/m^2^. Exclusion criteria: (1) severe cardiac insufficiency, hepatic or renal dysfunction, other major systemic diseases; (2) preoperative body temperature >37.5 °C; and (3) long-term use of sedative or analgesic medications. These criteria were applied to minimize confounding factors and ensure patient safety, as pre-existing organ dysfunction, acute infection, or chronic exposure to sedatives could significantly alter the response to anesthetics and affect the interpretation of study outcomes.

**Figure 1:**
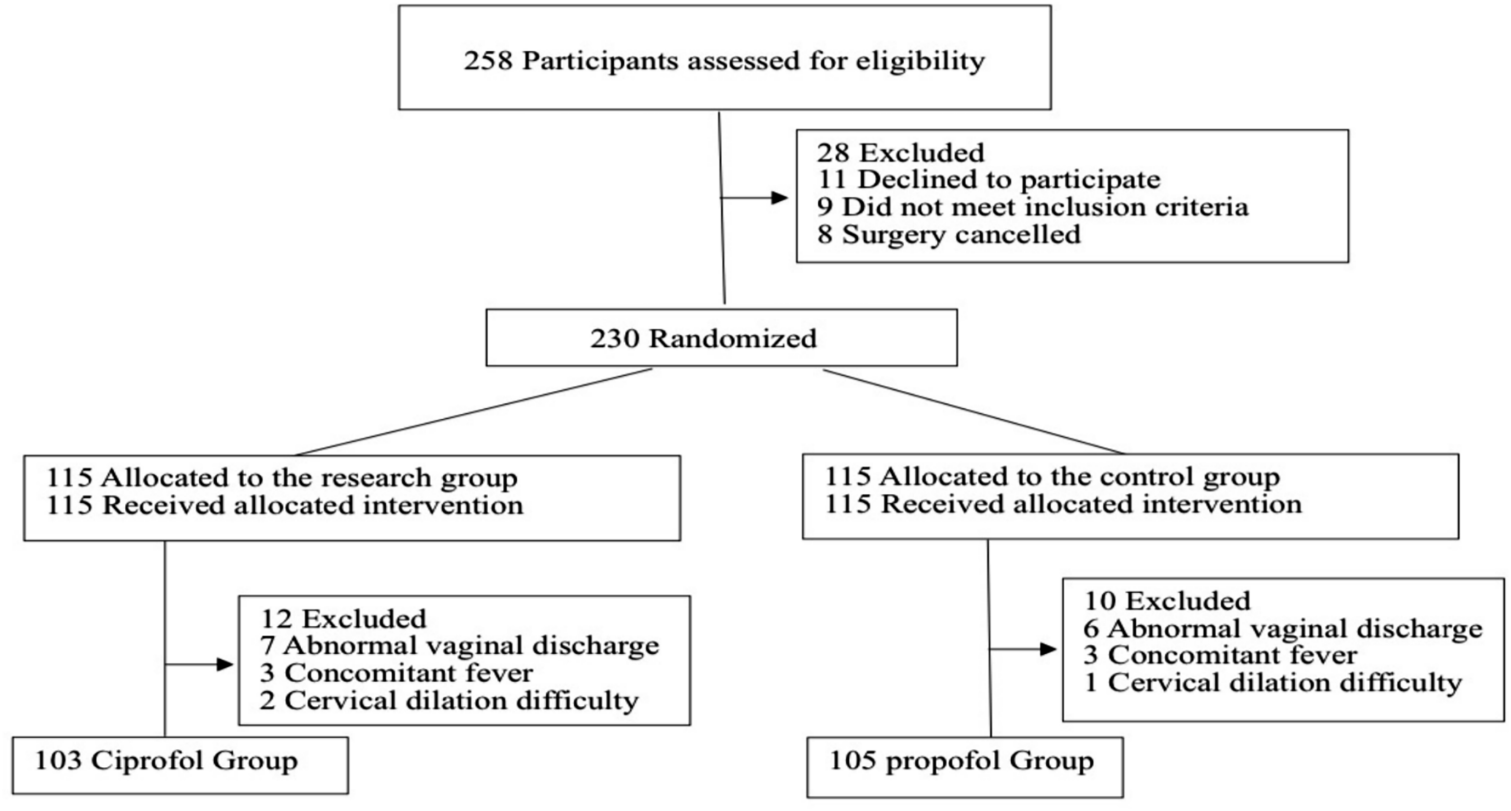
The flowchart of participants selection.

Sample size calculation was based on the incidence of hypotension. According to previous studies, ciprofol reduced the incidence of hypotension compared with propofol after anesthesia induction (RR: 0.63, 95% CI: 0.42 ~ 0.94, *p* = 0.02) (6). With a power of 1−*β* = 0.8, significance level *α* = 0.05, PASS 11 software estimated a required sample size of 208 patients. Considering a dropout rate of 10%, at least 230 participants was recruited. The primary outcome of this study was the incidence of hypotension. Patients were randomized into two groups by an intern not involved in subsequent research using SPSS 26.0 software: the ciprofol group (Group C, *n* = 103) and the propofol Group (Group P, *n* = 105) (Figure 1).

### 2.2 Anesthetic method

After standard fasting, patients were monitored for blood pressure (BP), electrocardiography (HR), pulse oxygen saturation (SpO₂), and BIS upon entering the operating room. All patients received intravenous alfentanil 10 μg/kg (7), followed by ciprofol 0.4 mg/kg in the ciprofol group or propofol 2.0 mg/kg in the propofol group over 10 ~ 15 s. A laryngeal mask airway was inserted after induction, and volume-controlled mechanical ventilation was initiated with oxygen flow at 2 L/min, inspiratory-to-expiratory ratio of 1:2, tidal volume 6 ~ 8 mL/kg, respiratory rate 12 ~ 14 breaths/min, end-tidal CO₂ maintained at 30 ~ 45 mmHg, and airway pressure at 12 ~ 20 cmH_2_O. Anesthesia was maintained with continuous infusion of ciprofol 0.8 mg/ (kg · h) or propofol 5 mg/ (kg · h), adjusted according to anesthetic depth. All surgeries were performed by the same experienced surgeon using standardized techniques. Patients were transferred to the post-anesthetic care unit after surgery.

### 2.3 Monitoring indicators

Vital signs: BP, HR, SpO, and BIS were observed and recorded for all patients at the following time points: Pre-anesthesia (T0), after anesthesia induction and laryngeal mask placement (T1), during cervical dilation (T2), during electrosurgical resection (T3), and at the end of surgery (T4). Total anesthetic dosage, recovery time, orientation recovery time, VAS scores for uterine contraction pain after awakening, and postoperative adverse events were recorded.

### 2.4 Statistical analysis

Data were analyzed using SPSS 26.0 software. Measurement data are presented as mean ± standard deviation ( $\bar{x}$ ±s). The *t*-test was used for comparisons between two groups. Repeated measures analysis of variance was used for multi-time point comparisons, followed by paired *t*-tests for within-group comparisons. Count data are presented as number [*n* (%)] and analyzed using the χ^2^ test. A *p*-value <0.05 was considered statistically significant.

## 3 Results

### 3.1 Comparison of basic characters of the two groups

There were no statistically significant differences between the two groups with respect to age, body weight, body mass index (BMI), ASA (I ~ II) and duration of surgery (*p* > 0.05) (Table 1).

**Table 1:** Basic characteristics of the two groups.

Variables	Group C (*n* = 103)	Group P (*n* = 105)	*P* value
Age (years)	24.7 ± 9.2	25.2 ± 9.1	0.083
Weight (kg)	67.4 ± 9.3	67.3 ± 9.7	0.216
BMI (kg/m^2^)	23.1 ± 3.2	23.2 ± 3.1	0.129
ASA (I/II) (*n*)	51/52	53/52	0.217
Duration of surgery (min)	25.4 ± 6.7	26.3 ± 6.5	0.143

ASA, American society of anesthesiology classification; BMI, body mass index.

### 3.2 Comparison of MAP, HR, and BIS between the two groups

Preoperative baseline values of mean arterial pressure (MAP), heart rate (HR), and BIS did not differ significantly between the two groups (*p* > 0.05). In the group P, MAP, HR, and BIS values at T1, T2, and T3 were all significantly decreased compared with baseline (T0) (*p* < 0.05). In the group C, BIS values at T2 and T3 were significantly lower than baseline (T0) (*p* < 0.05), whereas no significant change was observed at T4 (*p* > 0.05) (Table 2).

**Table 2:** Comparison of MAP, HR, and BIS between the two groups.

Item	Group C (*n* = 103)	Group P (*n* = 105)	*P* value
MAP			
T0	81.6 ± 6.3	82.9 ± 5.7	0.812
T1	76.1 ± 6.5	64.2 ± 5.4	0.014*
T2	75.4 ± 5.1	70.1 ± 5.3	0.023*
T3	78.5 ± 5.4	71.5 ± 5.7	0.025*
T4	81.5 ± 4.3	81.7 ± 3.6	0.243
HR			
T0	85 ± 17	84 ± 15	0.117
T1	81 ± 16	71 ± 13	0.023*
T2	74 ± 9	71 ± 7	0.032*
T3	76 ± 7	72 ± 8	0.031*
T4	82 ± 11	83 ± 9	0.351
BIS			
T0	97.7 ± 0.5	96.9 ± 0.3	0.402
T1	45.1 ± 4.5	45.8 ± 5.2	0.124
T2	40.4 ± 4.3	44.3 ± 4.9	0.031*
T3	40.2 ± 5.1	45.3 ± 4.7	0.023*
T4	83.3 ± 4.0	84.9 ± 5.1	0.134

MAP, mean arterial pressure; HR, heart rate; BIS, Bispectral Index System. * Denotes significant difference between the two groups.

### 3.3 Comparison of total dosage of study drug, recovery time, and orientation recovery time

The total dosage of anesthetic agents was significantly higher in the propofol group than in the ciprofol group (*p* < 0.05). Recovery time and orientation recovery time were significantly longer in the ciprofol group compared with the propofol group (*p* < 0.05) (Table 3).

**Table 3:** Comparison of total dosage of study drug, recovery time, and orientation recovery time of the two groups.

Item	Group C (*n* = 103)	Group P (*n* = 105)	*P* value
Total dosage of study drug (mg)	35.4 ± 5.3	165.1 ± 8.7	0.017^*^
Recovery time (min)	7.2 ± 2.1	4.7 ± 1.9	0.031^*^
Orientation recovery time (min)	11.2 ± 5.1	7.3 ± 5.2	0.043^*^

*Denotes significant difference between the two groups.

### 3.4 Comparison of after-awakening uterine contraction pain

**Figure 2:**
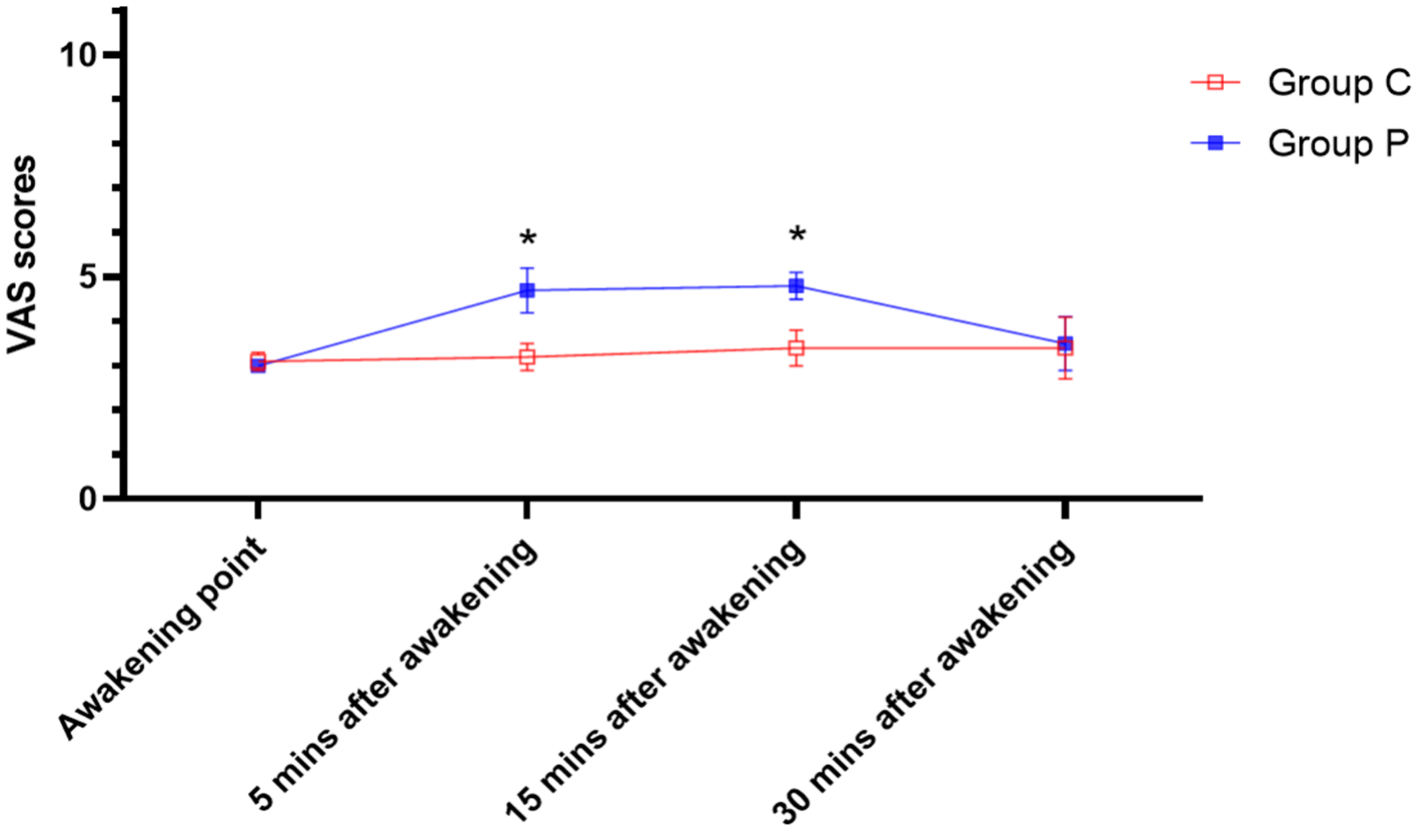
Comparison of after awakening uterine contraction pain. *Denotes significant difference between the two groups.

At 5 and 15 min after awakening, the visual analogue scale (VAS) scores for uterine contraction pain were significantly lower in the ciprofol group than in the propofol group (*p* < 0.05). At 30 min after awakening, no statistically significant difference in VAS scores was observed between the two groups (*p* > 0.05) (Table 4; Figure 2).

**Table 4:** Comparison of VAS scores at the awakening point, 5 min, 15 min, and 30 min after awakening of the two groups.

Item	Group C (*n* = 103)	Group P (*n* = 105)	*P* value
Awakening point	3.1 ± 0.2	3.0 ± 0.1	0.513
5 min after awakening	3.2 ± 0.3	4.7 ± 0. 5	0.027^*^
15 min after awakening	3.5 ± 0.3	4.6 ± 0.2	0.021^*^
30 min after awakening	3.4 ± 0.7	3.5 ± 0.6	0.643

*Denotes significant difference between the two groups.

### 3.5 Comparison of adverse events

The incidence of injection pain was significantly lower in the ciprofol group than in the propofol group (*p* < 0.001). Within 30 min after awakening, there were no statistically significant differences between the two groups in the incidence of dizziness, postoperative nausea and vomiting (PONV), or hypoxemia (SpO₂ < 95%) within 30 min after awakening (*p* > 0.05) (Table 5).

**Table 5:** Comparison of adverse events between the two groups.

Post-procedural AE	Group C (*n* = 103)	Group P (*n* = 105)	*P* value
Injection pain	3 (2.9)	49 (46.7)	<0.001*
Dizziness	13 (12.6)	12 (11.4)	0.524
PONV	5 (4.9)	7 (6.7)	0.681
Hypoxemia	4 (3.9)	5 (4.8)	0.713

AE, adverse events; PONV, postoperative nausea and vomiting. *Denotes significant difference between the two groups.

## 4 Discussion

Compared with routine diagnostic hysteroscopy, hysteroscopic transcervical resection of submucosal myoma is a more complex and time-consuming procedure. High-risk factors include excessive uterine flexion, cervical stenosis, prior cervical surgery, intrauterine adhesions, inappropriate selection of electrosurgical instruments, and limited surgical experience (8). In addition, intraoperative electrosurgical manipulation may trigger vagal reflexes, increasing the complexity of anesthetic management (9). Insufficient depth of anesthesia during hysteroscopic electrosurgery may result in pronounced pain perception, leading to patient movement that interferes with surgical manipulation and increases the risk of complications such as uterine perforation and injury to adjacent organs, including the bowel and bladder (10, 11).

Propofol is widely used for its rapid onset, fast metabolism, and minimal drug accumulation. However, its major limitations include respiratory depression and cardiovascular suppression. Ciprofol is an (R)-configured small-molecule compound and a short-acting GABA_A_ receptor agonist. Its anesthetic effect is mediated by enhancement of GABA receptor–mediated influx, producing sedation and hypnosis. Structural modification of propofol by introducing a cyclopropyl group confers chirality and enhances steric interaction with the GABA_A_ receptor, thereby increasing receptor affinity (12). As a result, ciprofol demonstrates rapid onset, fast recovery, and reduced injection pain, making it a promising intravenous anesthetic agent (13).

Alfentanil, a fentanyl derivative and selective μ-opioid receptor agonist, provides potent analgesia with minimal impact on myocardial oxygen supply and hemodynamics and a relatively low incidence of postoperative respiratory depression (14). The combination of ciprofol or propofol with alfentanil satisfies the requirement for rapid postoperative recovery following hysteroscopic procedures and is commonly used in clinical anesthesia practice (15).

For induction of general anesthesia, the recommended initial dose of ciprofol does not exceed 0.4 mg/kg and should be administered slowly via intravenous injection. Ciprofol provides rapid induction, stable hemodynamics, and minimal intubation response and injection pain (16). For maintenance of anesthesia, a continuous infusion starting at 0.8 mg/(kg·h) is recommended, with dose adjustments based on patient response. Infusion rates between 0.4 and 2.4 mg/(kg·h) generally achieve adequate anesthetic depth, with stable BIS monitoring and a low incidence of hypotension (17). In elective surgeries lasting less than 2 h, the average recovery time after discontinuation of ciprofol is typically within 10 min (12, 18).

Regarding adverse effects, ciprofol demonstrates a safety profile similar to that of propofol, with a trend toward lower incidences of respiratory events, hypotension, and bradycardia (19). In the present study, decreases in MAP and HR after anesthesia induction were significantly greater in the propofol group than in the ciprofol group (*p* < 0.05), indicating that ciprofol exerts less hemodynamic suppression, consistent with previous clinical studies (20, 21).

The total anesthetic dosage was significantly higher in the propofol group than in the ciprofol group (*p* < 0.05). Recovery time and orientation recovery time were longer in the ciprofol group, consistent with findings reported by Akhtar et al., whose meta-analysis demonstrated that time to full alertness was significantly longer with ciprofol compared with propofol (MD: 0.93 min; 95% CI: 0.28–1.58; I^2 = 68%; *p* < 0.01) (22). Additionally, ciprofol has been shown to exert a supra-additive synergistic interaction with opioids, which may potentiate sedative depth and prolong emergence and recovery, as reflected by prolonged time to eye-opening and return of orientation (23).

In addition, VAS scores at 5 and 15 min after awakening were significantly lower in the ciprofol group (*p* < 0.05), indicating superior postoperative analgesia. These findings align with previous studies demonstrating that ciprofol provides more effective relief of uterine contraction pain than propofol (24, 25).

Injection pain remains a persistent clinical challenge associated with propofol administration, with reported incidence rates ranging from 28 to 90% during injection via dorsal hand veins (26). Injection pain may negatively affect patient experience and anesthesia quality. Ciprofol has been shown to significantly reduce injection pain and improve patient satisfaction (27). In the present study, the incidence of injection pain and other adverse events was significantly lower in the ciprofol group than in the propofol group (*p* < 0.05).

Study Limitations: First, this was a single-center study, which may limit the generalizability of the findings. Second, although the sample size was calculated *a priori*, the relatively modest sample size precludes subgroup analyses (e.g., based on BMI or fibroid size). Third, the anesthesiologists were not blinded to group allocation due to the distinct characteristics of the two anesthetics, potentially introducing performance bias. Lastly, while we observed a longer recovery time with ciprofol, the clinical significance of this difference (a few minutes) in the context of day-surgery settings warrants further investigation. Future multicenter, large-scale randomized controlled trials are warranted to address these limitations and further determine the optimal dosages of ciprofol for hysteroscopic surgery.

Furthermore, while the present study focused on clinical efficacy and safety, future research should incorporate cost-effectiveness analyses to inform healthcare resource allocation. Additionally, the availability of ciprofol may vary by region; multicenter studies across diverse geographical settings would help validate the generalizability of our findings and assess its real-world applicability.

## 5 Conclusion

Ciprofol combined with alfentanil provides effective sedation, reduces injection pain, maintains more stable hemodynamics, and lowers the incidence of respiratory-related adverse events during hysteroscopic transcervical resection of submucosal myoma.

## Data Availability

The original contributions presented in the study are included in the article/supplementary material, further inquiries can be directed to the corresponding author.
